# Spread of the Zoonotic Nematode *Baylisascaris procyonis* into a Naive Raccoon Population

**DOI:** 10.1007/s10393-023-01655-6

**Published:** 2023-11-16

**Authors:** Mike Heddergott, Stéphanie Lippert, Annette Schliephake, Wolfgang Gaede, Anna Schleimer, Alain C. Frantz

**Affiliations:** 1 Musée National d’Histoire Naturelle, 25 rue Muenster, L-2160 Luxembourg, Luxembourg; 2 Department for Veterinary Medicine, State Institute for Consumer Protection of Saxony-Anhalt, Haferbreiter Weg 132-135, 39576 Stendal, Germany; 3 Fondation Faune Flore, 24 rue Muenster, L-2160 Luxembourg, Luxembourg

**Keywords:** Genetic structure, Invasive species, Parasitic nematode, Range expansion, Public health, Zoonosis

## Abstract

The raccoon roundworm (*Baylisascaris procyonis*), a gastrointestinal nematode of the raccoon (*Procyon lotor*), may cause a severe form of *larva migrans* in humans, which can lead to death or permanent neurological damage. Although roundworms were inadvertently introduced to Europe alongside their raccoon hosts, the parasite is not present in every raccoon population. It is important to understand the geographic distribution of *B. procyonis*, as early and rapid treatment can prevent severe pathologies in humans. We present evidence for the roundworm spreading into a naive raccoon population through natural dispersal of infected raccoons. We sampled 181 raccoons from Saxony-Anhalt, a German federal state containing contact zones of different raccoon populations, two of which were previously free of the parasite. We screened the raccoons for roundworms and used microsatellite-based assignment tests to determine the genetic origin of the raccoons and their parasites. We detected roundworms in 16 of 45 raccoons sampled in a previously roundworm-free area in the northern part of the state. The largest proportion of the genetic ancestry (≥ 0.5) of the 16 raccoon hosts was assigned to the previously naive raccoon population. Conversely, the genetic ancestry of almost all the roundworms was assigned to the nearest roundworm population in the southern part of the state. Infected raccoons have, therefore, spread to the north of the state, where they interbred with and infected local raccoons. It seems likely that the roundworms will continue to spread. Health authorities should consider continuous surveillance programmes of naive populations and raise public awareness.

**Supplementary Information:**

The online version contains supplementary material available at 10.1007/s10393-023-01655-6.

## Introduction

The introductions of animals into a new range increase the risk of inadvertently translocating exotic pathogens alongside the host, endangering human and animal health, biodiversity, and the natural environment (Daszak et al. 2000; Taraschewski, 2006; Zhang et al. 2022). Helminths have a widespread distribution, and many hosts are afflicted with multiple species of parasitic worms. They will thus frequently be translocated alongside their hosts (Taraschewski, 2006; Gilabert and Wasmuth 2013). Once established, alien helminths can become highly invasive and cause serious disease in naive, native free-living animals and humans (Kirk, 2003; Taraschewski, 2006; Barratt et al. 2016; Kołodziej-Sobocińska et al. 2016).

The raccoon roundworm (*Baylisascaris procyonis*) is a gastrointestinal nematode of the raccoon (*Procyon lotor*). The parasite can be very common in its native Central and North American range, where sometimes > 75% of a study population is infected (Kazacos, 2016). As primary hosts, raccoons can release millions of roundworm eggs via their faeces. It has been estimated that faeces contain > 1.6 × 10^4^ eggs per gramme of faecal material (Reed et al. 2012) and that a single infected raccoon can contaminate 0.03 ± 0.01 ha/year with *B. procyonis* eggs (Ogdee et al. 2017). The eggs become infective within 11–14 days and, since they withstand both sub-zero and high temperatures and tolerate different soil textures and moistures, they can survive in the environment for years (Page et al. 2011; Shafir et al. 2011; Ogdee et al. 2016).

Infection with *B. procyonis* in raccoons is usually harmless. After ingestion of infective eggs, the hatched larvae move into the raccoon’s small intestine wall where they mature into adult worms. However, in non-definitive hosts, the growing larvae undertake an aggressive somatic migration (called *larva migrans*), which can be fatal if they invade the central nervous system (Sorvillo et al., 2002). The roundworm shows low specificity: Over 130 vertebrate species with clinical larval infections caused by the nematode have been identified (Page, 2013).

Humans can become accidental hosts of the raccoon roundworm and develop baylisascariasis with severe symptoms. The clinical severity of an infection depends on the number of eggs ingested and the primary site of larval migration (Gavin et al. 2005; Wise et al. 2005). Patients may suffer from visceral, ocular, or neural *larva migrans*. In the case of the latter, the larvae migrate to the central nervous system, which, in the absence of effective treatment, leads to death or permanent neurological sequelae (Wise et al. 2005). Exposure usually occurs at raccoon latrines, and infants are particularly at risk from faecal–oral transmission, as are people with pica or geophagia syndromes (Strausbaugh et al. 2004). Occupational contact with raccoons and raccoon latrines in or near residential property may also lead to an increased risk of infection (Conraths, 1996; Sorvillo et al. 2002; Sapp et al. 2018).

As a result of joint introduction, both the raccoon and its roundworm parasite are present in Europe (Heddergott et al. 2020). Raccoons are particularly widespread in Germany. Over the past three decades, their abundance and distribution has increased substantially, and they are predicted to be present in most parts of the country by mid-century (Fischer et al. 2016). They are also spreading into urbanised areas where they come into closer contact with people and their pets (Hohmann and Bartussek 2018; Louvrier et al. 2022). Human baylisascariasis seems to be rare: So far, only one non-fatal case (Küchle et al. 1993) and four seropositive people (Conraths, 1996) have been reported from Germany in the literature. Nevertheless, given the severity of the clinical cases reported from North America, the disease is of public health importance (Wise et al. 2005). The World Health Organization has classified baylisascariasis as a zoonosis ‘with current and potential increasing impact’ in Europe (Anonymous, 2004). It is thus necessary to monitor raccoon populations for the presence of the roundworm as early recognition and rapid treatment may prevent severe pathologies in humans (Muganda et al. 2018).

**Figure 1:**
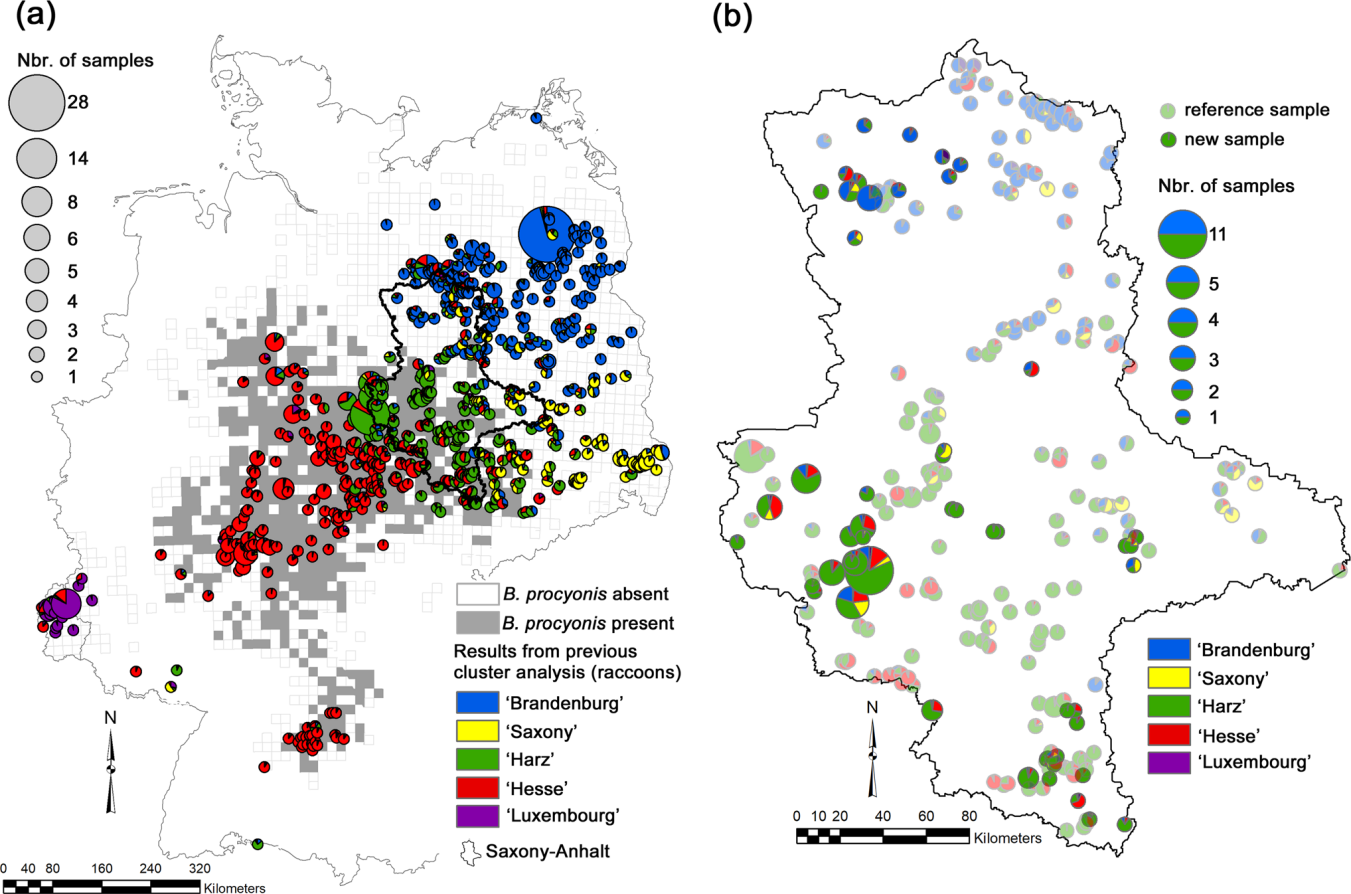
Population genetic structure of raccoons (*Procyon lotor*) in Germany and Saxony-Anhalt. **a** The spatial extent of the five main STRUCTURE clusters inferred by Frantz et al. (2021) and comparison of the geographic distribution of the raccoon roundworm (*Baylisascaris procyonis*). The roundworm data are based on the analysis of 8184 raccoons (Heddergott et al. 2020), and the presence/absence of the parasite is plotted using the 10 × 10-km ETRS89-LAEA5210 EEA reference grid. Different colours represent different genetic populations. **b** Population genetic structure of raccoons in Saxony-Anhalt. The 85 new samples were analysed together with 859 reference individuals in programme STRUCTURE, with the number of genetic clusters (*K*) set to *K* = 5. In both figures, the sizes of the pie charts are proportional to the number of individuals investigated from a specific locality. For further methodological details, please refer to the Material and Methods section.

Based on the analysis of their genetic structure, raccoon populations in Germany have emerged from at least five separate founder events. Fischer et al. (2015) and Frantz et al. (2021) showed distinct genetic clusters in Luxembourg/western Germany, in Hesse and adjacent areas (Central Germany), around the Harz Mountains (Central Germany), in Brandenburg and adjacent areas (northeastern Germany), and in Saxony (eastern Germany; Fig. 1a). So far, the occurrence of the roundworm has only been confirmed for the populations in Hesse and the Harz Mountains, the remaining populations were free of *B*. *procyonis* (Heddergott et al. 2020). The close correspondence between the spatial extent of these two genetic populations and the spatial distribution of the parasite (Fig. 1a) suggested that the occurrence of *B*. *procyonis* is due to infection of the founder individuals (Frantz et al. 2021). However, because of a large amount of genetic admixture in contact zones between populations (Fig. 1a), Frantz et al. (2021) suggested a likely spread of the nematode into non-infected populations in the near future.

The federal state of Saxony-Anhalt, located in eastern–Central Germany, contains contact zones of all four Central and eastern German raccoon populations (Fig. 1). Raccoons in the north and the east of the federal state have been free of the roundworm with the raccoons in the north mostly belonging to the ‘Brandenburg’ population and the eastern raccoon mostly to the ‘Saxony’ population (Heddergott et al. 2020; Frantz et al. 2021; Fig. 1). In the present study, raccoons from Saxony-Anhalt were screened for the presence of the roundworm, and the genetic origin of the raccoon hosts and parasites was determined. We conducted this work with the aim of detecting evidence for the spread of the raccoon roundworm into a naive raccoon population.

## Methods

### Sample Collection

**Figure 2:**
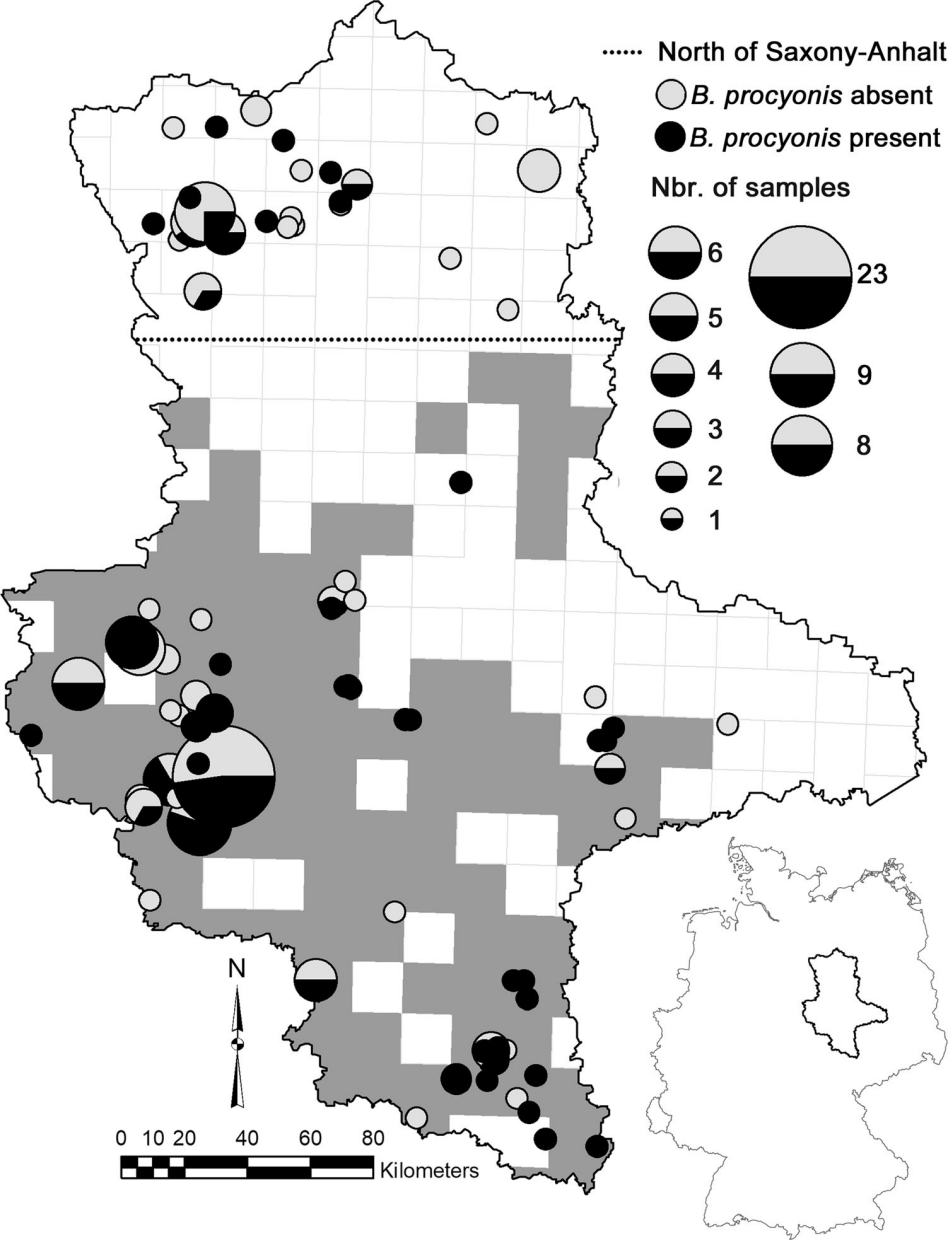
Geographic distribution and infection status of the 181 raccoons (*Procyon lotor*) from Saxony-Anhalt sampled for this study. We refer to the samples above the dotted line as being from ‘the north of Saxony-Anhalt’. Inset: location of Saxony-Anhalt within Germany.

Between May 2020 and April 2021, we collected 181 harvested raccoons from Saxony-Anhalt (Fig. 2). We investigated the presence of *B. procyonis* worms by macroscopic analysis of the intestinal contents. We stored the worms and a piece of muscle tissue from each raccoon host in 96% absolute ethanol. The Wilson score interval was used to calculate the 95% confidence intervals of the proportion of infected animals in the sampled population (Newcombe, 1998).

### Laboratory Work

The DNeasy 96 blood and tissue kit (Qiagen, Hilden, Germany) was employed to extract DNA from one large (> 3 cm) roundworm per raccoon following the manufacturer’s instructions. We digested a roughly 1-cm-long fragment of the worm and eluted the extracted DNA in a final volume of 50 μl. DNA from the raccoon tissue samples was extracted using an ammonium acetate-based salting-out method (Miller et al. 1998). DNA extracts were quantified with a Drop-Sense 16 spectrophotometer (Trinean, Gentbrugge, Belgium). Following the methods outlined by Osten-Sacken et al. (2018), we generated microsatellite-based genetic profiles for the roundworms (14 loci) and their raccoon hosts (17 loci).

### Population Genetic Analyses: Raccoons

Frantz et al. (2021) used Bayesian clustering methods to analyse the population genetic structure of a raccoon reference dataset consisting of 859 tissue samples from animals sampled across their German range (Fig. 1a). This dataset included 365 individuals that had already been analysed by Fischer et al. (2015), who did not find evidence for systematic deviations from Hardy–Weinberg proportions and linkage equilibria in the 17 loci used to generate the raccoon genetic profiles. The clustering analysis of the large dataset by Frantz et al. (2021) confirmed the presence of the five clusters (‘Brandenburg’, ‘Harz’, ‘Hesse’, ‘Luxembourg’, and ‘Saxony’; see Introduction and Fig. 1a) inferred by Fischer et al. (2015). We used the results of the STRUCTURE v.2.3.4 (Pritchard et al. 2000) clustering analysis (number of distinct genetic clusters *K* = 5) by Frantz et al. (2021) in the present analysis and referred to it as ‘reference cluster analysis’.

The genetic profiles of raccoons sampled for the present study, and the 859 reference samples were genotyped in the same laboratory using the same protocols. This allowed us to combine both datasets to determine the genetic and geographic origin of the infected raccoons. To exclude the possibility that the infected individuals collected for the present study originated from a separate introduction event (Maas et al. 2021), we used GENECLASS 2.0.g (Piry et al. 2004) to calculate the probability of the new animals belonging to each of the five major clusters inferred by Frantz et al. (2021). We calculated these exclusion probabilities based on the Monte Carlo method of Paetkau et al. (2004) and simulated 10,000 multi-locus genotypes. The five reference populations were created based on the reference cluster analysis by modally assigning each of the 859 reference samples to the cluster for which it had the highest inferred ancestry coefficient *q*. Similarly to other ecological studies that identify genetic immigrants, we used an exclusion threshold of *p* < 0.01 (Frantz et al. 2017).

We then analysed the pooled dataset with STRUCTURE conducting ten independent runs each for a number of distinct genetic clusters *K* varying between 1 and 20. In order to assign the animals to one the five major clusters (‘Brandenburg’, ‘Harz’, ‘Hesse’, ‘Luxembourg’, and ‘Saxony’), we focussed on the assignment values obtained for *K* = 5. In order to further exclude the possibility that the infected individuals originated from a separate introduction event (i.e. that these animals formed a distinct STRUCTURE cluster), we also verified the assignment results generated with the STRUCTURE run with the highest log-likelihood values. The parameters for the STRUCTURE analyses were based on Osten-Sacken et al. (2018). We used the admixture model with correlated allele frequencies and a uniform prior for ALPHA, the Dirichlet parameter for the degree of admixture, which was allowed to vary between clusters. However, to improve convergence, we performed 10^6^ Markov chain Monte Carlo (MCMC) iterations after a burn-in of 10^6^ iterations. After accounting for label switching and confirming the lack of multimodality, the proportion of membership of each individual was averaged over replicate runs.

### Population Genetic Analyses: Raccoon Roundworm

Osten-Sacken et al. (2018) analysed the population genetic structure of raccoon roundworms in Central Germany. They used the same 14 microsatellites as the present study, showing that the loci did not systematically deviate from Hardy–Weinberg and linkage equilibria. They provided evidence for the presence of two genetic clusters whose boundaries corresponded to the boundary between two raccoon populations (Hesse and Harz). The new roundworm genetic profiles generated here, and the 226 profiles of Osten-Sacken et al. (2018) were also genotyped in the same laboratory using the same protocols. We were thus able to pool both datasets to determine the genetic and geographic origin of the roundworms infecting raccoons in Saxony-Anhalt.

Similarly to the raccoons, we first assigned the 226 reference roundworms to one of the two clusters (‘Harz’ and ‘Hesse’) based on the STRUCTURE *K* = 2 ancestry coefficients calculated by Osten-Sacken et al. (2018). We then used GENECLASS to calculate the probability of each of the new roundworms belonging to one of the two clusters, using the same parameters as above. We then analysed the pooled dataset with STRUCTURE, conducting ten independent runs of *K* = 1–6, using the same parameters as above. In order to assign the animals to one the two previously identified roundworm clusters (‘Harz’ and ‘Hesse’), we focussed on the assignment values obtained for *K* = 2. To exclude the possibility that the infected individuals originated from a separate introduction event, we also verified the assignment results generated with the STRUCTURE runs with the highest log-likelihood values.

All maps were generated with ArcMap v.10.3 (ESRI Inc., Redlands, California). The roundworm records of Heddergott et al. (2020) obtained by analysing 8184 raccoons, we plotted onto the 10 × 10-km ETRS89-LAEA5210 reference grid of the European Environment Agency.

## Results

*Baylisascaris procyonis* was detected in 88 of the 181 raccoons (48.6%, 95% CI 41.4%–55.6%) sampled in Saxony-Anhalt. We detected the parasite in almost all parts of the federal state. In the north of the state (Fig. 2), we detected the roundworm in 16 out of 45 raccoons (35.6%, 95% CI 23.2%–50.2%) in an area that was previously considered free of *B. procyonis* and was inhabited predominantly by raccoons assigned to the Brandenburg cluster (Fig. 1).

We generated a genetic profile of at least 13 microsatellite loci for 85 of the 88 infected raccoons. None of these 85 animals could be excluded at the *p* < 0.01 threshold from all five reference populations (Table S1). When analysing the pooled dataset, the log-likelihood values of six of the ten *K* = 5 STRUCTURE runs converged on a higher value (Fig. S1), and we considered the ancestry coefficients *q* of these six runs only. The STRUCTURE analysis suggested that most of the 16 infected raccoons sampled in this area had mixed ancestry (Fig. 1b). Nevertheless, the ancestry of ten animals was assigned to the Brandenburg cluster with *q* ≥ 0.500 (range: 0.500 ≤ *q* ≤ 0.897; Fig. 1b). In nine of these ten animals, the second highest *q* value was obtained for the Harz cluster (0.066 ≤ *q* ≤ 0.324).

In the STRUCTURE analysis where *K* varied between 1 and 20, there was no clear support for a specific number of clusters (Fig. S1). The highest log-likelihood values (*K* = 7–10) converged poorly. When mapping the ancestry coefficients of the *K* = 9 run with the highest overall log-likelihood estimate, we did not find evidence for some or all of the 85 new samples forming a distinct genetic cluster, even though most of the newly-inferred clusters were in part located in Saxony-Anhalt (Fig. S2). At *K* = 9, STRUCTURE inferred a distinct cluster in southern Germany and split the Brandenburg cluster into two geographically relatively coherent clusters. The remaining two clusters were mostly located in and around Saxony-Anhalt and overlapped with existing clusters.

**Figure 3:**
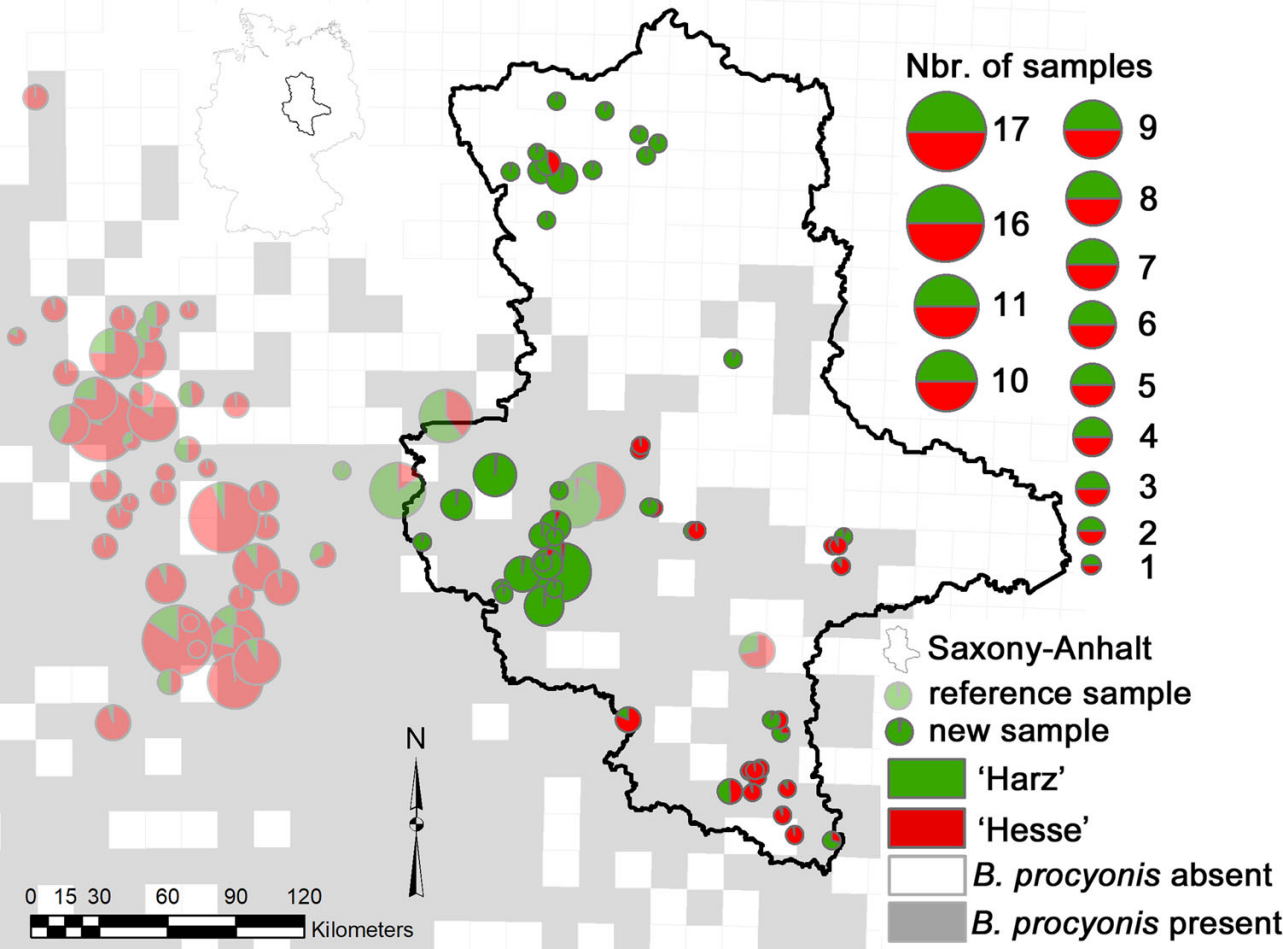
Population genetic structure of the raccoon roundworm (*Baylisascaris procyonis*) in Saxony-Anhalt and neighbouring regions. The 88 new samples were analysed together with 226 reference individuals in programme STRUCTURE, with the number of genetic clusters (*K*) set to *K* = 2, following Osten-Sacken et al. (2018). The background grid, based on the 10 × 10-km ETRS89-LAEA5210 EEA reference grid, indicates the presence/absence of the parasite, based on the analysis of 8184 raccoons (Heddergott et al. 2020; see also Fig. 1). Different colours represent different genetic populations, and the sizes of the pie charts are proportional to the number of individuals investigated from a specific locality. For further methodological details, please refer to the Material and Methods section. Inset: location of Saxony-Anhalt within Germany.

We generated a complete 14 loci-based genetic profile for 88 roundworms (each from a different raccoon). One roundworm (from the southwest of Saxony-Anhalt) was excluded at the *p* < 0.01 threshold from both reference populations (Table S2). When analysing the pooled dataset, the log-likelihood values of the ten *K* = 2 STRUCTURE runs all converged (Fig. S3). Altogether 62 of the 88 new samples (70.5%) were assigned to the Harz reference cluster with *q* ≥ 0.932 (Fig. 3). Fifteen of the 16 roundworms sampled in the north of Saxony-Anhalt were assigned to the Harz cluster with *q* ≥ 0.951. In the complete STRUCTURE analysis where *K* varied between 1 and 6, the highest log-likelihood values (that also converged) were obtained for *K* = 4 (Fig. S3). When mapping the ancestry coefficients for *K* = 4, one of the newly identified clusters overlapped strongly with the Hesse cluster, while a third cluster was formed by a smaller number of roundworms in the east of our study area (Fig. S4). However, 15 of the 16 roundworms sampled in the north of Saxony-Anhalt were still assigned to the equivalent of the *K* = 2 Harz cluster with *q* ≥ 0.919.

## Discussion

Although apparently a rare event, infection with the raccoon roundworm can be fatal in humans. It is thus necessary to monitor raccoon populations for the presence of the parasite as early recognition and rapid treatment can prevent severe pathologies in humans. Here, we provide evidence for *B. procyonis* spreading into a previously raccoon roundworm-free population in northern Saxony-Anhalt because of natural dispersal of infected animals.

Heddergott et al. (2020) and Frantz et al. (2021) found a close correspondence between the spatial extent of the Harz and Hesse raccoon genetic populations and the spatial distribution of the raccoon roundworm. These authors concluded that the occurrence of the roundworm in a raccoon population was due to the infection of the founder individuals. Consequently, Heddergott et al. (2020) could not provide evidence for roundworm occurrence in northern Saxony-Anhalt, whose raccoons were predominantly assigned to the Brandenburg population by Frantz et al. (2021). Until 2018, no raccoon roundworm had been detected in the Brandenbourg population (Heddergott et al. 2020). The present study provided clear evidence for the expansion of the parasite into this previously raccoon roundworm-free area, as we detected 16 infected raccoons in the north of the federal state. The corresponding prevalence estimate of 35.6% was lower than the estimate obtained for the state as a whole, but the 95% confidence interval of both estimates overlapped. Moreover, the estimate was in line with prevalence values reported for many North American populations (Kazacos, 2016), and those obtained for 69 German administrative districts (interquartile range 34.4%–49.7%) where the parasite was present, and > 25 raccoons had been sampled (Heddergott et al. 2020). The prevalence estimate obtained for northern Saxony-Anhalt was thus comparatively high, given that the area was roundworm-free until recently.

The infected raccoons in northern Saxony-Anhalt were mostly genetically admixed, but in almost all cases, the largest proportion of their genetic ancestry was assigned to the Brandenburg cluster, followed by the Harz cluster. Thus, it seems that infected raccoons from the Harz population, which occupies most of Central and southern Saxony-Anhalt (Fig. 1), spread to the north of the state, where they interbred with and infected local raccoons from the Brandenburg population. Consistent with this conclusion is the finding that the genetic ancestry of the roundworm in northern Saxony-Anhalt was assigned to the Harz roundworm cluster. The results of the population genetic analyses of the raccoons and the roundworms thus both indicated that the roundworms were transmitted to the Brandenburg population by raccoons originating from the Harz population.

The log-likelihood values inferred by STRUCTURE suggested that we may have underestimated the true number of genetic populations for both raccoons (*K* = 5) and roundworms (*K* = 2). However, the additional clusters often seem to be statistical artefacts rather than biologically meaningful. In the case of the raccoon, convergence of the STRUCTURE runs was poor, especially at higher values of *K*, and in both species, the newly derived clusters often overlapped geographically. Artificial clusters can result from deviations from random mating that is not caused by genetic discontinuities, such as a gradient of isolation-by-distance (Frantz et al. 2009) or the presence of related individuals (Anderson and Dunham 2008). Importantly, uncertainty about the exact nature of the population genetic structure in both the raccoon host and the parasite did not alter our conclusion that roundworms from the Harz genetic population have infected a previously naive population.

## Conclusion

In the present study, we provided evidence for the spread of the raccoon roundworm into a previously uninfected raccoon population. Transmission occurred at the ‘distribution edge’ of the roundworm-free population, through dispersal from a nearby, infected population. It remains to be seen whether the parasite will spread to the ‘core’ of the Brandenburg population. Given our current results, this is quite possible. Controlling the spread of *Baylisascaris procyonis* in German raccoons is like to be difficult, if not impossible, due to high raccoon densities, high prevalence of the parasite in raccoons, and the potential for transmission to other animals. Given that early detection and rapid treatment can prevent severe pathologies in humans, health authorities should consider continuous surveillance of the Brandenburg population for roundworms, while raising awareness of this public health problem.

### Supplementary Information

Below is the link to the electronic supplementary material.

Supplementary file1 (DOCX 1111 KB)

## Data Availability

The datasets generated and/or analysed during the current study are available from the corresponding author on reasonable request.
