# Supplementary material for: Spread of the Zoonotic Nematode Baylisascaris procyonis into a Naive Raccoon Population
Source: Ecohealth. 2023 Nov 16;20(3):263–72. doi: 10.1007/s10393-023-01655-6 (PMC10757695; doi:10.1007/s10393-023-01655-6)
Supplement: Supplementary file 1 — Supplementary file1 (DOCX 1111 KB) [file 10393_2023_1655_MOESM1_ESM.docx]

Table S1: Probabilities of the 85 infested raccoons sampled in Saxony-Anhalt originating from one of the five German reference populations, calculated by means of exclusion tests with the GENECLASS software. Results are shown for the ten individuals with the lowest overall probabilities. For further information on methodologies, please refer to the Material and methods section.

| Sample | Latitude | Longitude | Reference population | | | | |
| --- | --- | --- | --- | --- | --- | --- | --- |
|  |  |  | Brandenburg | Harz | Hessen | Luxembourg | Saxony |
| M1008 | 51.606437 | 11.128528 | <0.001 | 0.034 | 0.047 | <0.001 | 0.004 |
| M1038 | 51.702619 | 12.299023 | 0.042 | 0.054 | 0.001 | <0.001 | 0.038 |
| M1010 | 51.752752 | 12.287350 | <0.001 | 0.087 | 0.008 | <0.001 | 0.002 |
| M1009 | 51.925756 | 10.935173 | 0.042 | 0.106 | 0.100 | <0.001 | 0.012 |
| M1080 | 52.800115 | 11.368017 | 0.217 | 0.018 | 0.002 | <0.001 | 0.002 |
| M1089 | 52.744346 | 11.502342 | 0.271 | 0.275 | 0.032 | <0.001 | 0.114 |
| M1072 | 52.694020 | 11.530495 | 0.307 | 0.251 | 0.061 | <0.001 | 0.065 |
| M1007 | 51.648277 | 10.960836 | 0.002 | 0.315 | 0.010 | <0.001 | 0.090 |
| M1084 | 51.774851 | 11.171207 | 0.013 | 0.365 | 0.046 | <0.001 | 0.147 |
| M1017 | 51.688745 | 11.197901 | 0.008 | 0.388 | 0.021 | <0.001 | 0.060 |

Table S2: Probabilities of the 88 raccoon roundworms sampled in Saxony-Anhalt originating from one of the two German reference populations, calculated by means of exclusion tests with the GENECLASS software. Results are shown for the ten individuals with the overall lowest probabilities. For further information on methodologies, please refer to the Material and methods section.

| Sample | Latitude | Longitude | Reference population | |
| --- | --- | --- | --- | --- |
|  |  |  | Harz | Hessen |
| M1045 | 51.327394 | 11.460114 | <0.001 | 0.006 |
| M1042 | 51.201936 | 11.940250 | <0.001 | 0.012 |
| M1094 | 51.789453 | 11.729364 | <0.001 | 0.013 |
| M1087 | 51.206883 | 11.978531 | <0.001 | 0.036 |
| M1000 | 51.150602 | 11.860514 | <0.001 | 0.039 |
| M1088 | 51.201936 | 11.959305 | <0.001 | 0.054 |
| M1056 | 51.708802 | 11.139300 | 0.147 | 0.176 |
| M1069 | 51.148179 | 11.948490 | 0.008 | 0.187 |
| M1059 | 52.701458 | 11.100655 | 0.153 | 0.306 |
| M1004 | 51.774851 | 12.308207 | 0.339 | 0.345 |


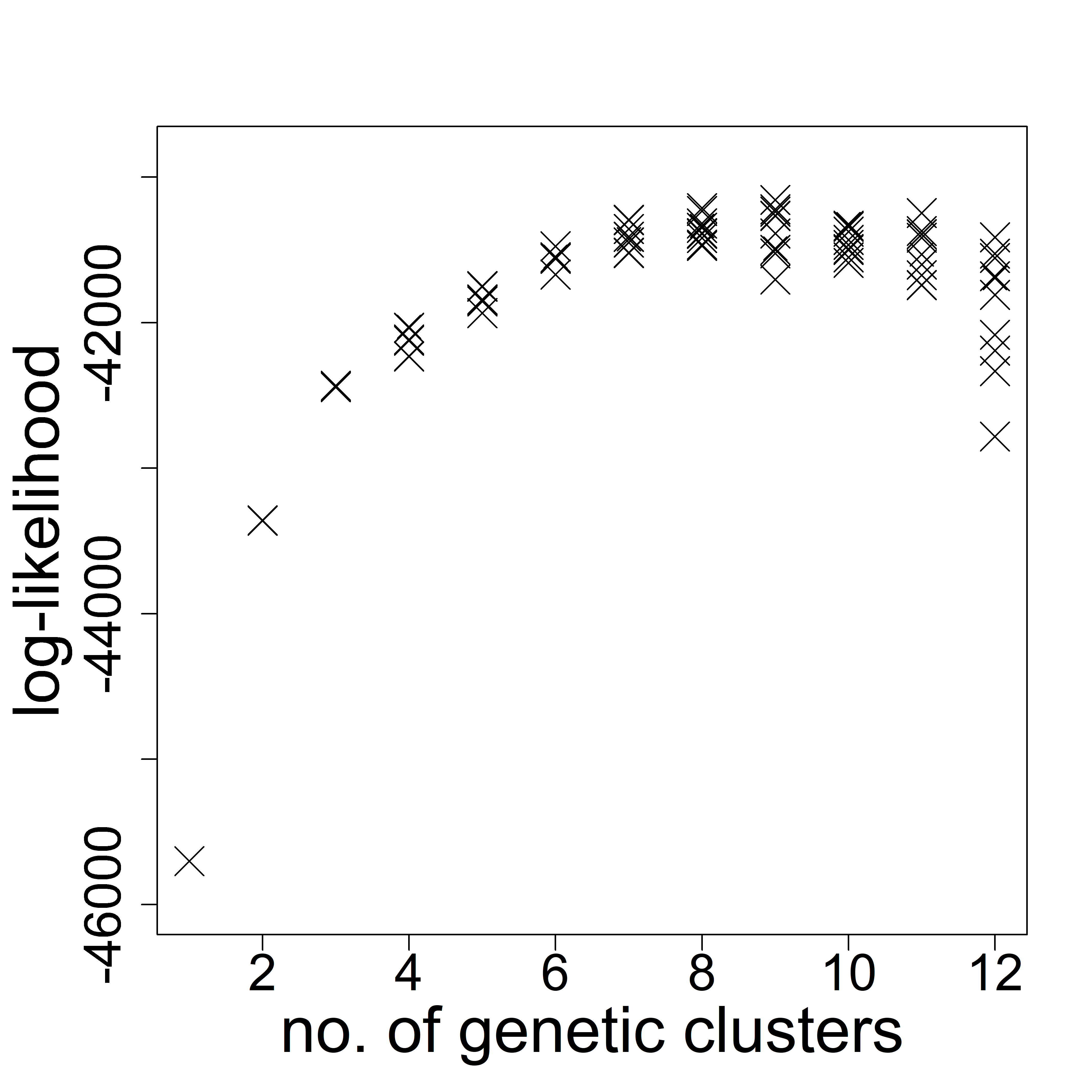


Fig. S1: Plot of the number of STRUCTURE clusters tested against their estimated log-likelihood for the complete raccoon dataset. Please refer to the Material & Methods section for further information on the parameters using for the analysis.


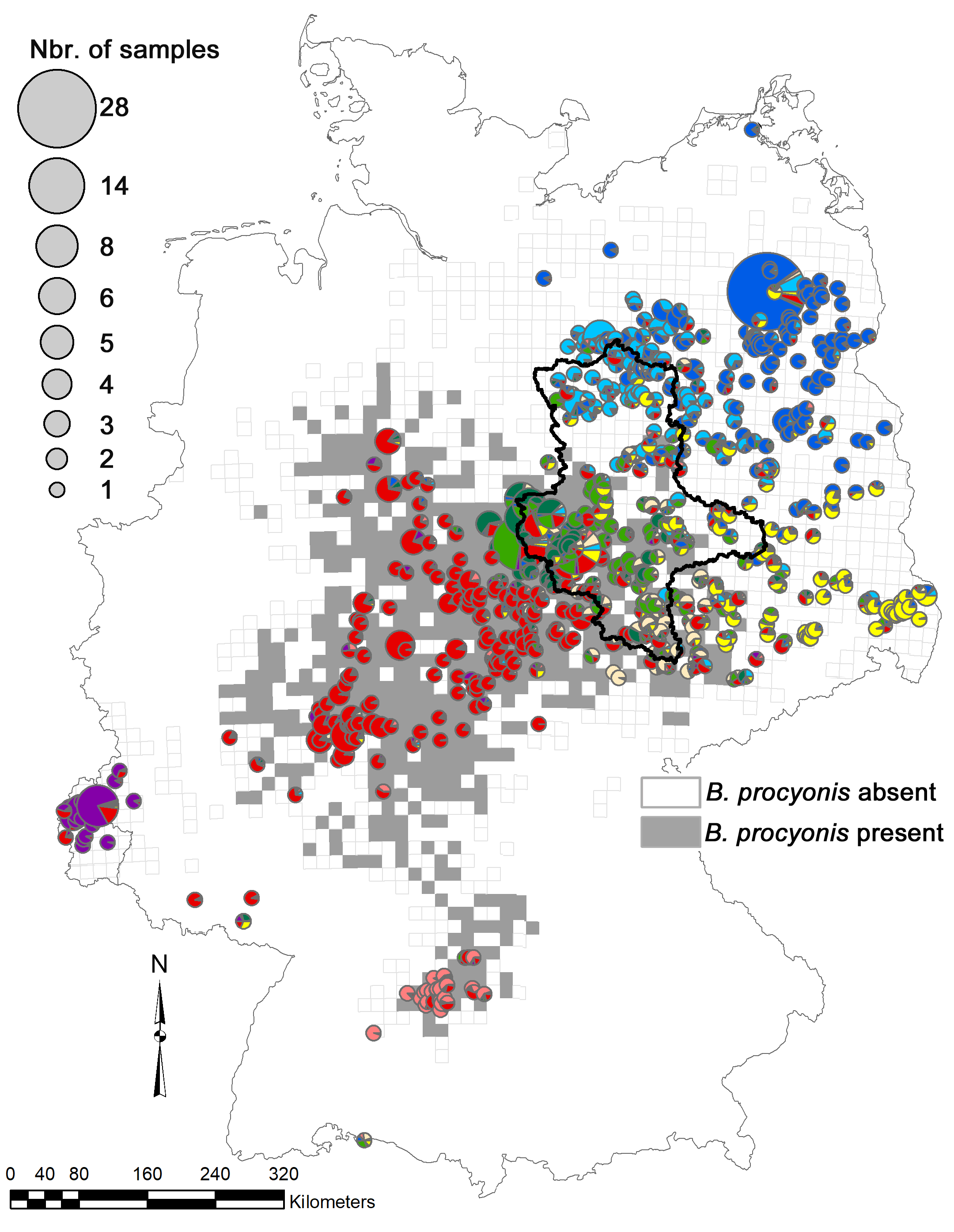


Fig. S2: Geographic distribution of nine raccoon genetic clusters inferred by the STRUCTURE run with the highest overall log-likelihood estimate. STRUCTURE was run with the number of the genetic clusters *K* varied between 1-20. Different colours represent different genetic populations and the size of the pie chat is proportional to the number of individuals investigated from a specific locality. The background grid, based on the 10x10 km ETRS89-LAEA5210 EEA reference grid, indicates the presence/absence of the parasite, based on the analysis of 8,184 raccoons (Heddergott et al. 2020; see also Fig. 1). For further methodological details, please refer to the Material and Methods section.


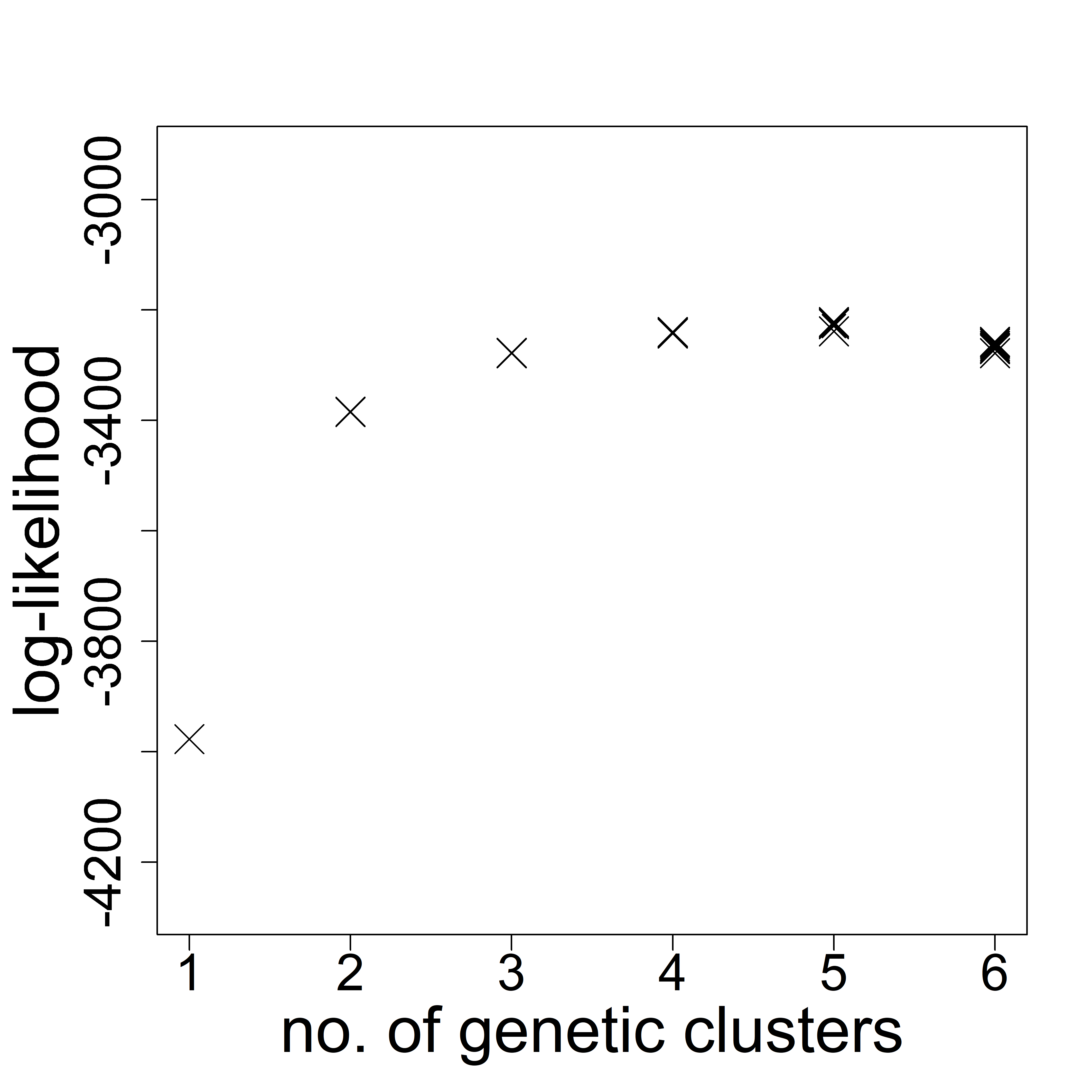


Fig. S3: Plot of the number of STRUCTURE clusters tested against their estimated log-likelihood for the complete roundworm dataset. Please refer to the Material & Methods section for further information on the parameters using for the analysis.


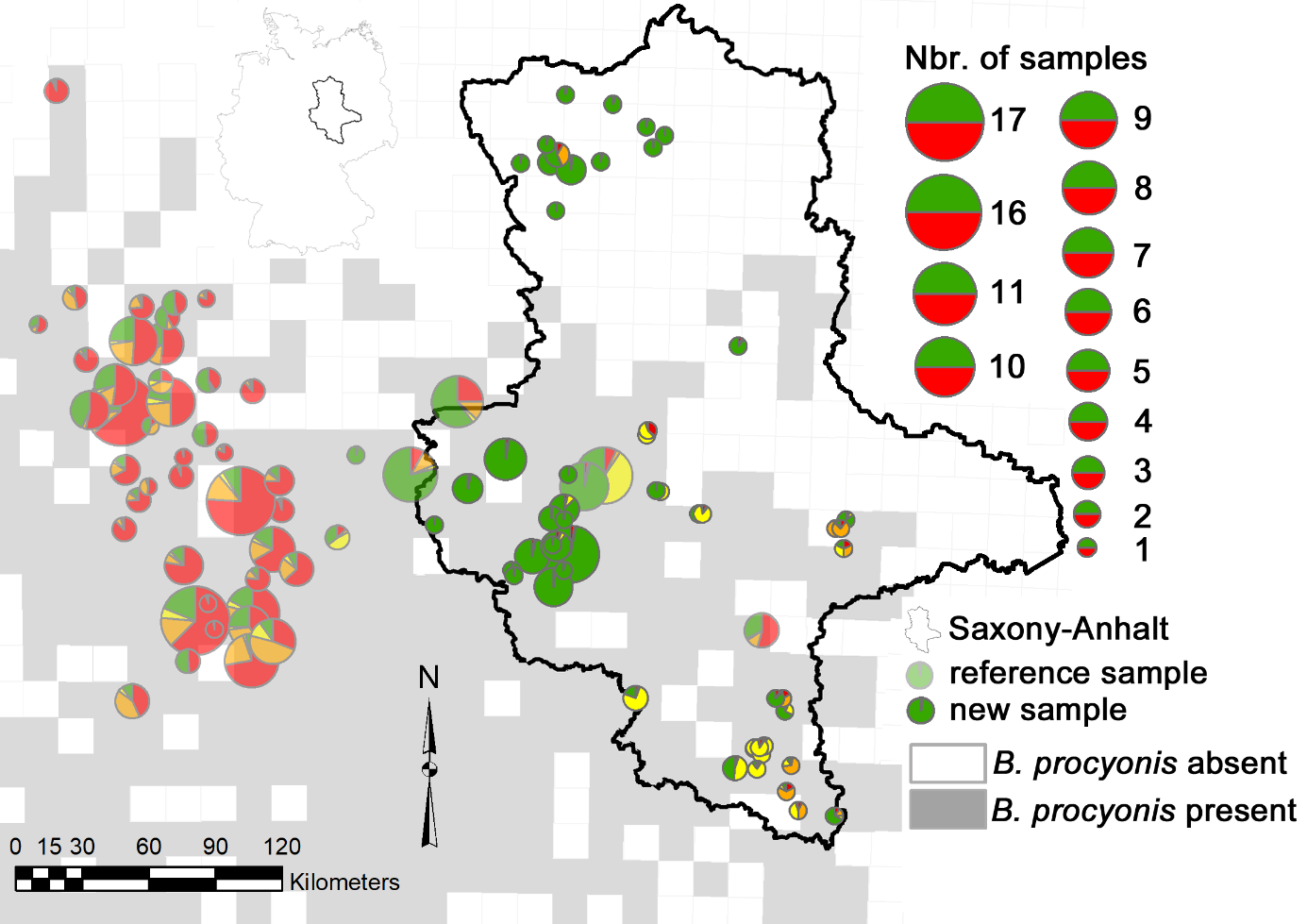


Fig. S4: Geographic distribution of four roundworm genetic clusters in Saxony-Anhalt and neighbouring regions inferred by STRUCTURE for *K*=4. Different colours represent different genetic populations and the size of the pie chat is proportional to the number of individuals investigated from a specific locality. The background grid, based on the 10x10 km ETRS89-LAEA5210 EEA reference grid, indicates the presence/absence of the parasite, based on the analysis of 8,184 raccoons (Heddergott et al. 2020; see also Fig. 1). For further methodological details, please refer to the Material and Methods section. Inset: location of Saxony-Anhalt within Germany.
